# SG-APSIC1108: Implementation of infection prevention and control in Indonesian hospitals: Identification of strengths, gaps, and challenges in current practices

**DOI:** 10.1017/ash.2023.52

**Published:** 2023-03-16

**Authors:** Indri Rooslamiati Supriadi, Leli Saptawati, Nani H. Widodo, Hindra Irawan Satari, Gortap Sitohang, Yuslely Usman, Ida Bagus Anom, Ratih Dian Saraswati, Juliëtte A. Severin

**Affiliations:** Ministry of Health of Indonesia, University Medical Centre Utrecht and Department of Medical Microbiology and Infectious Diseases, Erasmus University Medical Centre, Rotterdam, The Netherlands; Department of Microbiology, Faculty of Medicine, Universitas Sebelas Maret, Surakarta, Indonesia and Department of Microbiology, Moewardi Teaching Hospital, Surakarta, Indonesia, Indonesia; Directorate of Referral Health Care, Ministry of Health of Indonesia and National Working Group of Infection Prevention and Control, Ministry of Health of Indonesia, Jakarta, Indonesia; Cipto Mangunkusumo Hospital, Ministry of Health of Indonesia, Department of Child Health, Faculty of Medicine Universitas Indonesia, Jakarta, Indonesia, and National Working Group of Infection Prevention and Control, Ministry of Health of Indonesia, Jakarta, Indonesia; National Working Group of Infection Prevention and Control, Ministry of Health of Indonesia, Cipto Mangunkusumo Hospital, Ministry of Health of Indonesia, Jakarta, Indonesia; Jakarta, Center for Financing and Decentralization Policy Health Resources, Health Development Policy Agency, Ministry of Health of Indonesia, Jakarta, Indonesia; Directorate of Referral Health Care, Ministry of Health of Indonesia and National Working Group of Infection Prevention and Control, Ministry of Health of Indonesia, Jakarta, Indonesia; Center for Health Policy on Resilience System and Resource, Health Development Policy Agency, Ministry of Health of Indonesia, Jakarta, Indonesia; Department of Medical Microbiology and Infectious Diseases, Erasmus University Medical Centre, Rotterdam, The Netherlands

## Abstract

**Objectives:** Infection prevention and control (IPC) in hospitals is key to safe patient care. Currently, no data are available regarding the implementation of IPC in hospitals in Indonesia. We assessed the existing IPC practices in a nationwide survey using the World Health Organization (WHO) IPC assessment framework tool (IPCAF) to identify strengths, weaknesses, and challenges. **Methods:** A cross-sectional study was conducted from July to November 2021. Of all general hospitals in Indonesia, 475 (20%) were selected using stratified random sampling based on class (ie, A, B, C, and D; A being the larger hospitals with ≥250 beds) and region. IPCAF was translated into Indonesian and was tested in 4 hospitals. Questions were added regarding challenges in the implementation of IPC. Introduction meetings were held online with all selected hospitals, after which the IPCAF was sent as an online questionnaire. **Results:** In total, 355 hospitals (74.7%) participated in this study. The overall median score of IPCAF was 632.5. The level of implementation of IPC was mostly advanced (56.9%), followed by intermediate (35.8%), basic (7.0%), and inadequate (0.3%). The core component with the highest scores was IPC guidelines; almost all hospitals had guidelines on the most important topics, including hand hygiene. Core components with the lowest scores were surveillance of healthcare-associated infections (HAIs), education and training, and multimodal strategies. Although >90% of hospitals indicated that surveillance of HAIs was performed, 57.2% reported no availability of adequate microbiology laboratory capacity to support HAI surveillance. The most reported challenges in the implementation of IPC were behavior change and lack of availability of antibiograms. **Conclusions:** The implementation of the IPC core components in most Indonesian hospitals was “advanced.” For surveillance of HAIs, the need for the availability and capability of the microbiology laboratory was revealed.

